# Histopathological and imageological studies on clinical outcomes of mineralized collagen reconstruction rod for femoral head necrosis with one case report

**DOI:** 10.1093/rb/rbx013

**Published:** 2017-04-27

**Authors:** Baogang Xie, Hao Wang, Jianhua Hao, Ping Wang, Na Zhang, Jingjing Wu, Zhiye Qiu, Fuzhai Cui

**Affiliations:** 1 Department of Orthopaedics, 254th Hospital of PLA, No. 60 Huangwei Road, Hebei District, Tianjin 300142, China; 2 School of Materials Science and Engineering, Tsinghua University, Haidian District Beijing 100084, China

**Keywords:** femoral head necrosis, mineralized collagen, reconstruction rod for femoral head necrosis

## Abstract

In this article, the biodegradation process and bone formation of a mineralized collagen reconstruction rod embedding in necrosis of human femoral head were investigated by imageological and histological methods. Computed radiography (CR) computerized tomography (CT), common pathological section and hard tissue section analysis were used to evaluated the dynamics of imageological and histopathological changes of femoral head, interface between the host bone and implant and the bone reconstruction process. The results showed that the density of rods increased closed to that of host bones after 1 year implanting, and the interface between them turns to blurring. Hard tissue grinding sections analysis showed osteocytes appearing in sparse bone trabecular and bone pit region, as well as a few vessels in the degraded dye powder matrix were noticed, indicating the new bone forming between the implants and host bones. Regular decalcified sections analysis showed scattered osteoclasts, multinucleated giant cells and fibrosis components existing in the degraded rod and the host bone trabecular. Degraded debris was endocytosed by giant cells, and vascular network formed around the boundaries of the implanted rod. The good osteointegration has been expressed by the interface between the implanted rod and the host bone becoming blurred. Histological results indicated that the implanted rod degradation process and new bones regeneration simultaneously occurred around the boundaries of embedding rod. New bone and host bone were hinged and co-existed.

## Introduction

Non-traumatic femoral head necrosis is a kind of multiple diseases in persons from 30 to 50 years old. For these patients, all possible endeavors have been made to stop the deteriorate process of femoral head and prevent the femoral head from collapsing, as such, saving their joints and delaying (or avoiding) the joint replacement surgery. Currently, for the early stage of the collapse of the femoral head, marrow core decompression (CD) and autologous bone grafting were usually performed, combining with structural support and blood supply reconstruction surgeries. With such treatments, tantalum or titanium rods, as well as autologous fibula with vascular pedicle graft, were used, which can provide structural support to the femoral head and prevent further collapse during the repairing period. However, these treatments have several deficiencies in clinical applications. Firstly, in necrotic bone area, the host bone could not form osteointegration with tantalum or titanium rod. The subchondral bone plate was still difficult to be repaired when the femoral head collapses was inevitable. Secondly, tantalum rod can only provide support for very limited necrosis area, position deviation of the tantalum or titanium rods during the implantation may affect their functionalities. Meanwhile, taking fibula to fill the necrotic region may also induce some complications, such as the movement in the weakness, ankle discomfort and calf parts and feeling of lower limb disabled. The incidence of ankle and lower limb pain increased with the time. In addition, by free fibula transplantation, proximal femur would face the potential risk of bone fracture. Moreover, grafting tantalum or titanium rods in the femoral head and femoral neck may cause the vascular changing and bring difficulties in artificial joint replacement surgery [1]. Therefore, the new materials with good biomechanical properties and biological activity were needed to treat the femoral head necrosis clinically.

Mineralized collagen is a type of artificial biomimetic biomaterial with similar chemical composition and microstructure to the natural bone [2, 3], which mainly composed of collagen fibrils and nano-sized hydroxyapatite. During the formation of the mineralized collagen, hydroxyapatite nucleated and grew on the surface of the collagen fibrils, such mineralization process is similar to the formation of bone [4]. Therefore, the biomimetic mineralized collagen has good biocompatibility, osteogenesis ability and osteoconductivity, which has been demonstrated by many animal experiments and clinical evidences [5–7]. In previous clinical applications, mineralized collagen has been used for treating necrosis of femoral head and achieved good outcomes [8]. Recently, the mineralized collagen was fabricated into a reconstruction rod with high strength to meet the requirements for the treatment of necrosis of femoral head. The reconstruction rod possesses high mechanical properties to provide enough biomechanical support for load-bearing site of human body. The density of the rod is ∼1.7 g/cm^3^, and the compressive strength is as high as ∼90 MPa [9], which are comparable to those of human cortical bone. Moreover, the compressive modulus of the rod is ∼1.8 GPa [9], which is a little lower than that of human cortical bone, resulting in no stress shielding as metallic materials.

Histopathological examination can directly reveal morphological changing of the tissue engineered artificial bone *in vivo* with their degradation and bone formation, but in clinical practice, there is quite few chance to take human histological specimen for pathological examination, yet only the imageological examination can be used to evaluate morphological changing of tissue engineered artificial bone with their degradation and bone formation indirectly. Although, in the animal experiments, the related histology and imageological study has been reported [10–12], but it cannot really reflect the embedded tissue**-**engineered artificial bone with its metabolic process *in vivo* due to differences between animal models and clinical presentation. However, there is no in-depth assessment of implanted collagen reconstruction rod using human histological specimen. In this study, one case accepted mineralized collagen reconstruction rod implantation after routine pulp CD for femoral head necrosis has been investigated, this patient accepted artificial total hip replacement due to the collapse of femoral head caused by over-weight and early weight bearing after surgery. We collected the removed femoral head and femoral neck for histopathological examination and explored correlation of histology and imageological changes after mineralized collagen reconstruction rod implantation. The results can provide histological basis for clinical application of imageological method to assess the degradation of tissue engineered artificial bone and new bone formation.

## Data and method

### Patient and clinical data

Male, 43-year-old, 110-kg weight, 20 years drinking experience (∼500 ml/day, alcohol by volume: 52%), irregular left hip pain and aggravated, with no radiating pain and effecting by weather changing, walking exacerbated the pain and no attenuating after resting. Computed radiography (CR)-reported trabecular bone at upper lateral region of left femoral head was no longer distinguish, computed tomography (CT) showed uneven increase of left femoral head bone density and effusion in the left hip cavity, magnetic resonance imaging (MRI) showed left femoral head bone T2W1 with marrow edema by fat suppression. Diagnosis: left femoral head avascular necrosis. According to the femoral head necrosis stage [13] in China, it was II b; According to China-Japan Friendship Hospital (CJFH) classification, it was L2; Harris score was 80 points. After hospital admission, marrow CD and implantation with the mineralized collagen reconstruction rod were carried out, which attenuated the pain symptoms effectively. However, due to premature walk of the patient, the symptoms came back again half year later and deteriorated gradually, limited left hip movement. X-ray, CT and MRI showed left femoral head with necrosis and collapses. According to the femoral head necrosis stages in China, it was IV b; According to CJFH classification, it was L2; Harris score was 65 points. After hospital admission, the artificial total left hip replacement was carried out 12 months after the primary surgery of marrow CD and mineralized collagen reconstruction rod implantation. The left femoral head was collected and fixed with 10% formalin for histopathological examination.

### Structure and mechanical properties of the material

**Figure 1:**
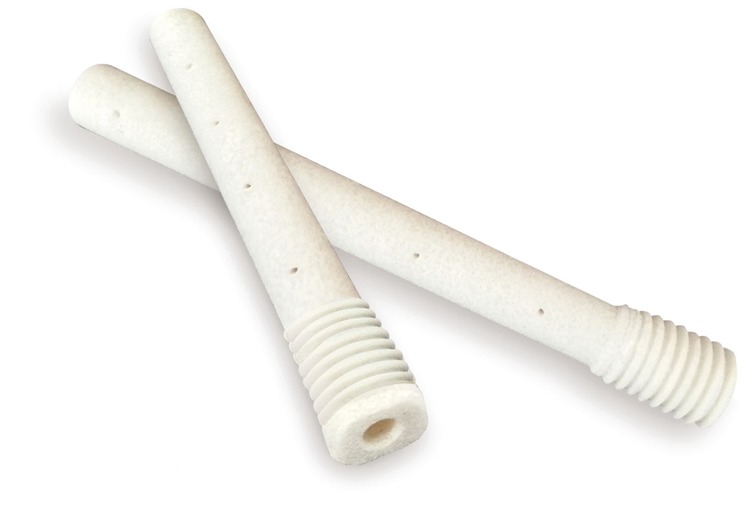
Reconstructional rod for femoral head necrosis

Reconstruction rod is made of the mineralized collagen with biomimetic human bone structure, which is a kind of commercial bone implant products “Bongold” (Beijing Allgens Medical Science and Technology Co., Ltd.) with external diameter of 10 mm and inner diameter of 3.5 mm, and the length is ranging from 85 to 110 mm with 5-mm interval (Fig. 1). The mineralized collagen was prepared by an *in vitro* biomimetic mineralization process that is similar to the formation of natural bone tissue as previously reported. In brief, water-soluble calcium salt solution and phosphate salt solution with Ca/P = 1.67 were added into acidic collagen solution to form MC deposition by adjusting pH value and temperature of the reaction system. The deposition was collected by centrifugation and freeze-drying, and then followed by a cold compression molding process with a certain pressure to achieve the results that appearance and density of the dense mineralized collagen were similar to those of natural cortical bone [2, 9].

### Pathological examination

In order to evaluate biodegradation of the implant material, osteointegration between the host bone and the implant, and formation of regenerated bone tissue, pathological examination including gross anatomical observation of host bone, reconstruction rod and the interface from coronal and transverse section were performed. Morphological observation and bio-compatibility evaluation of hard cross section of collum femurs and reconstruction rod was made by Germany EXAKT hard tissue cut and grinding system. Structural and cell morphological observation of coronal section of femoral head, collum femurs, and reconstruction rod interface were also performed. Under different amplification conditions, osteoclasts and lamellar bone trabecular formation, degradation products, changing process of multinucleated giant cells and fibrous components as well as formation of vessel network between the host bone and the implant were observed.

## Results

### Gross anatomy examination

**Figure 2:**
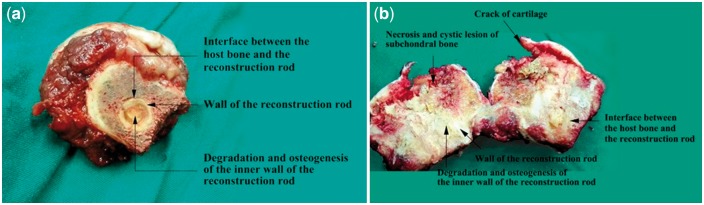
Gross anatomy of femoral head and collum femoris. (**a**) Transverse section for collum femoris; (**b**) coronal section for femoral head and collum femoris

The bone tissue at femoral neck region blended tightly together with mineralized collagen reconstruction rod. No space was found between host bone and reconstruction rod (Fig. 2), it was very difficult to separate the rod from host bone or take it out. The cartilage at the load-bearing region of femoral head was cracked, and a cystic cavity formed due to sub-cartilage bone necrosis.

### Hard tissue grinding section

**Figure 3:**
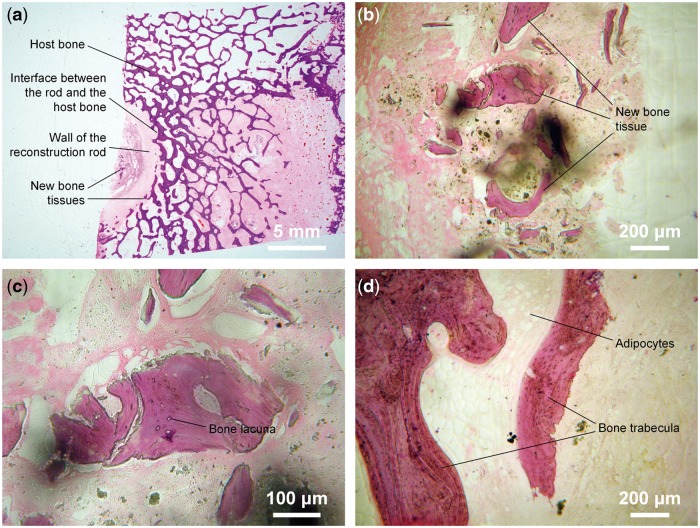
Hard tissue cutting and grinding sections at the interface between the reconstruction rod and the host bone. (**a**) Trabecular bone generated around outer edge of reconstruction rod companied by its degradation; (**b**) Pink-staining matrix is degradative implant with irregular shape and scattered cavity and trabecular bone; (**c**) Osteocytes in lacuna within lamellar trabecular bone; (**d**) Cancellous trabecular bone of the host bone and fat cells

As shown in Fig. 3, new trabecular bone generated and blended tightly with host bone at the boundaries of reconstruction rod. Osteocytes were found at bone lacuna, and some vessels appearing in the degraded pink-colored matrix, indicating that host bone showed no rejection response to embedded reconstruction rod.

### Decalcified paraffin section results

**Figure 4:**
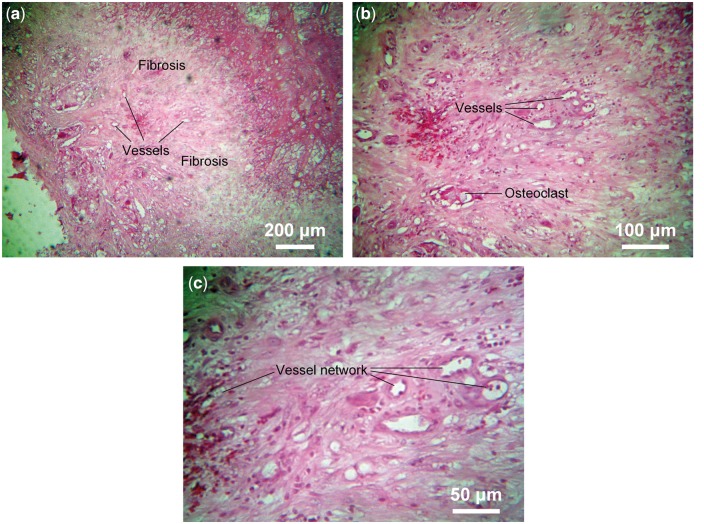
Decalcified paraffin sections at the interface between the reconstruction rod and the host bone. (**a**) The implant shown as uneven pink staining and mesh structure, which fused together with host bone and formed a curved and blurred boundary, and vessels can be seen in host bone but not implant; (**b**) Osteoclasts, vessel network and fibrosis in the border region of implant and host bone, phagocyte phagocytose debris of degradative implant; (**c**) Rich blood vessels network in the border region of host bone and implant

**Figure 5:**
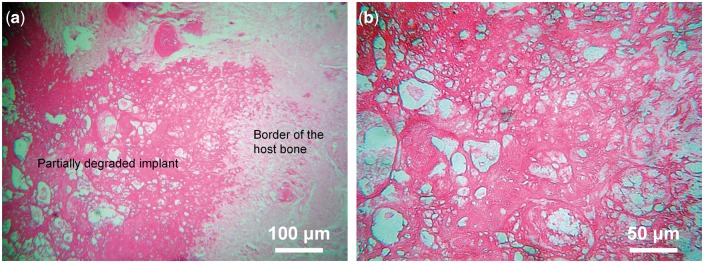
Decalcified paraffin sections of the regenerated bone tissues. (**a**) Implant shown as pink staining and partly degraded, rich small vessels scatter around host bone boundary; (**b**) Irregular size cavities scattered in pink staining implant

**Figure 6:**
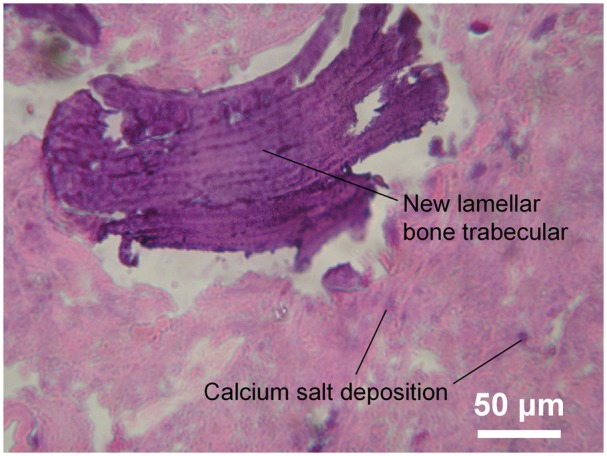
Degradation of the implant with calcium salt deposition and formation of bone trabecular

Host bone and implanted reconstruction rod fused together and formed a curved blurred boundary (Fig. 4a), at the border region, scattered rich vessel networks were observed. The vessels were with a diameter of 20–50 nm (capillary level), surrounded by a thin layer of epithelial cells (darker stained). Osteoclasts and fibrosis were also observed, and debris of degraded implant was phagocytosed by phagocytes (Fig. 4b and c). The partially degraded implant was shown pink staining and new small vessels could be observed near the border of the host bone (Fig. 5a), and the partially degraded implant was mesh structure with irregular size cavities (Fig. 5b). Rich lamellae bone-like tissue can be observed at the host bone side of border region with a size >100 nm. A gap region surrounded it can be visualized, which was due to the density difference between the new bone tissue and material when sectioning, commonly presented in de-calcified sample histology (Fig. 6).

### Imagology examination

**Figure 7:**
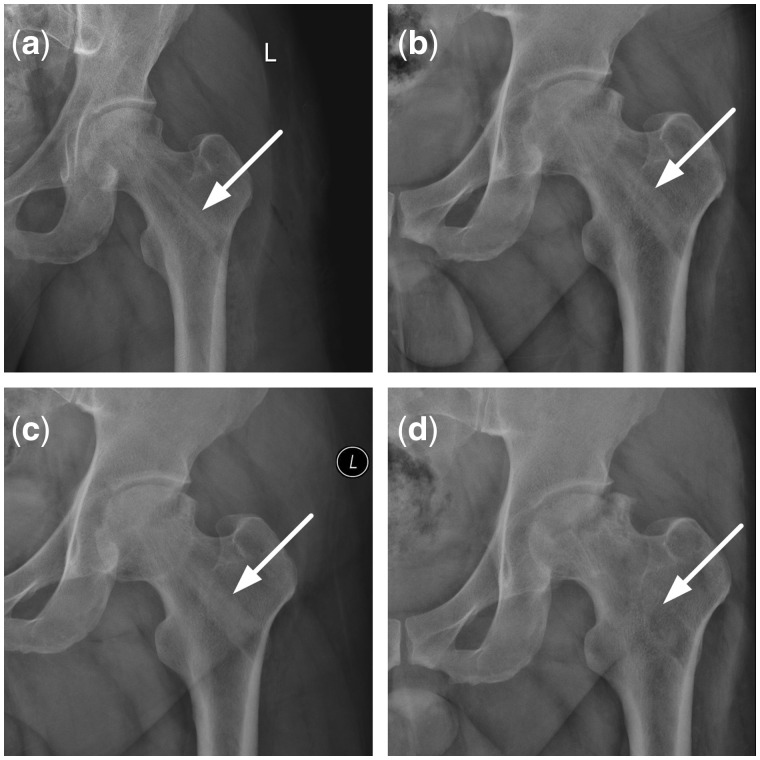
CR changes after implantation of the reconstructional rod. (**a**) CR in 3 days on postoperative; (**b**) CR in 3 months on postoperative; (**c**) CR in 5 months on postoperative; (**d**) CR in 12 months on postoperative

**Figure 8:**
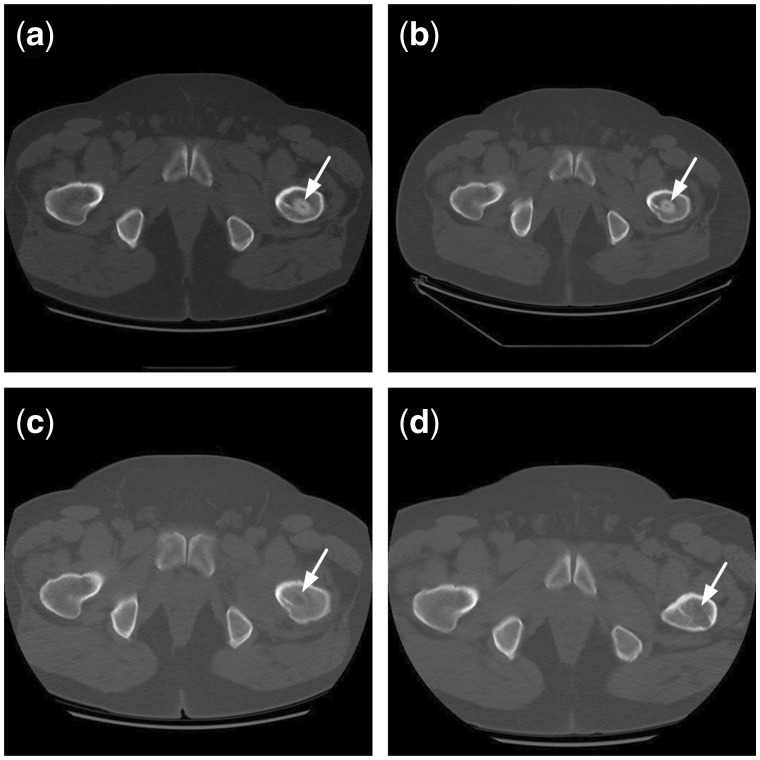
CT changes after implantation of the reconstructional rod. (**a**) CT in 3 months on postoperative; (**b**) CT in 5 months on postoperative; (**c**) CT in 9 months on postoperative; (**d**) CT in 11 months on postoperative

Both CR (Fig. 7) and CT (Fig. 8) showed that reconstruction rod contacted tightly with femoral head, femoral neck and intertrochanteric; however, the interface between the rod and host bone was very clear due to density difference between them after surgery. Twelve months later, the rod density reduced to similar to that of host bones, the interface between them became blurred. In addition, the inside diameter of rod looked bigger than that of after surgery.

### Imageological results are consistent with tissue pathological results

Implanted reconstruction rod has showed good bio-compatibility and no rejection by host bones. Tissue pathological examination shows new trabecular bone structure generating along with the degradation of reconstruction rod at the border region. Reconstruction rod contact host bone tightly without any gap. Imagology shows the interface between host bone tissue and reconstruction rod is blurred after surgery for 12 months, and reconstruction rod density decreases to much closer to the bone density, the insider diameter of reconstruction rod increases as well accordingly.

## Discussion

Femoral head necrosis is often occurring in patients aged 30–50 years old. For femoral head necrosis patients, in the early stage, retaining their femoral head is the preferred goal [14, 15]. Single hole CD with bone grafting is an effective treatment procedure for femoral head-preserving, which can reduce the loading pressure at femoral head, femoral neck and tuberosity obviously. Moreover, it is helpful to remove the necrotic tissue and create good bone graft bed for suppression of implantation. However, the large relief aperture range from 10 to 12 mm increases the risk of femoral head collapse and fracture at femoral neck and large tuberosity. Smith *et al.* [16] reported that the incidence of femoral neck fracture could reach 13% on early stage of intraoperative and postoperative CD. It has been reported that tantalum rod can effectively support femoral head and femoral neck, but with bone fractures caused by extraction of tantalum rods [17]. The animal experiments have confirmed that the tissue-engineered artificial bone rod, which is recently used in clinical, can promote the bone generation at femoral head and femoral neck region. But there are no tissue pathological research focused on human application. In this study, we use mineralized collagen reconstruction rod in ONFH repairing and analysis human histopathologic changing with imageological method. The results provide evidence to examine and assess the biological compatibility and bone metabolic process in histopathology.

Mineralized collagen as the bone repair material has widely been used in clinical, not only in the application for treating bone defects or bone nonunion, but also for repairing femoral head necrosis by changing its mechanical properties [13], which can effectively promote the repair process of femoral head necrosis owing to excellent mechanical properties, biodegradable and bone induction [6, 18–20]. In our previous work, both *in vitro* cell experiments and *in vivo* implantation assays demonstrated good biocompatibility of the mineralized collagen material. At day 7 of culture, osteoblast-like cells on the mineralized collagen material had proliferated 10 times than that of the seeding time point [9]. The non-degradation characteristics of metal rods used in clinical currently keep their interface with host bone permanently. The difference of elastic modulus between metal rods and host bones results in the interface loosening and rod falling out, thereby reducing the structural support on the femoral head and the restore effects of the decompression hole. These complications might be avoided by replacing the metal rod with mineralized collagen reconstruction rod, which is able to provide structural support at the early stage similar to the metal rod. As compared with pure calcium phosphate material, mineralized collagen has advantages in load bearing and energy dissipation ability when being implanted. Mechanical performance of bone is determined by the type and arrangement of different structural elements. Hard minerals, as bone hydroxyapatite, contribute to a high strength and stiffness to resist compressive stresses, while compliant collagen fibers provide high toughness and viscoelasticity to retard fracture propagation under shear or tensile stress [21, 22]. At same time, the mineralized collagen reconstruction rod could fuse the interface between the rod and the host bone through the degradation and osteogenesis process, the new bone tissue occupies the hole induced by CD. As such, disadvantages including fracture induced by large diameter of the decompression hole, rod falling out and unrepairable of the decompression hole could be solved. In clinical, imageological examination is usually used to indirectly evaluated degradation and osteogenesis process of mineralized collagen; however, no histopathological studies were reported till date.

The histological examination of the removed femoral bone and implantation of reconstruction rod indicated that (i) Due to the degradation and osteogenesis of the mineralized collagen reconstruction rod, the rod and the host bone fused via new bone formation. The femoral neck tissue and the reconstruction rod contacted tightly without any gap, it was difficult to remove the rod from the host bone. With the thickness of rod wall reducing, the inside diameter increased; (ii) The implant rod shows good biocompatibility and no tissue rejection action was found. There are renewable sparse bone trabecular in the implanting region, and osteocytes within bone pit Moreover, less vessel in degraded dye powder matrix can be observed, and bone trabecular structure integrated closely with the host bone on the edge of reconstruction rod, without gap and new bone forming; (iii) There are some trabecular bone on outer wall of the reconstruction rod. In the central area, there are osteoclast and lamellar bone trabecular formed during bone forming process. Osteoclasts, multinucleated giant cells and fibrosis were found scattered in the degrading and filling material with original trabecular bone. Multinucleated giant cells are devouring of the degrading material. On the boundaries of the implant, vascular networks are noticed. Yet lack of blood vessels inside the implanted part still exists. The histopathological examination showed the degradation and bone formation process conducted the inside and outside wall surfaces of the reconstruction rod; (iv) The interface between the host bone tissue and reconstruction rod becomes blurred. The density of reconstruction rod decreases to closer to the bone density with diameter of the reconstruction rod increasing; (v) Degradation of the reconstruction rod and bone formation replacement on radiographic show density decreasing gradually and the interface disappearing. Due to degradation of implant and new bone formation on both sides of the rod wall, the mechanical properties of the reconstruction rod must take mechanical support at early postoperative period. These factors can effectively prevent complications that lacking of bone repair ability and refunding rod with bone fracture occurred around the rod. CR and CT technology are used to observe reconstruction rod density, and interface changing between host bone and reconstruction rod, as well as the fusion degree between them and the structural support provided by the rod. This study provides histological basis for treating femoral head necrosis in clinical by using mineralized collagen reconstruction rod, as well as experimental method for further research.

## Conclusion

Owing to the degradation of the implanting rods, the density of the mineralized collagen became close to the host bone after the implant surgery for 1 year. Good osteointegration was observed expressing by the blurred interface between the rod and the host bone. Histological results indicated that the degradation process of the implants and regenerative process of new bone simultaneously occurred around the boundaries of embedded rod. New bone and host bone were hinged and co-existed.
